# The effectiveness of a real-world smoking cessation clinic: A prospective cohort study

**DOI:** 10.18332/tid/219278

**Published:** 2026-06-06

**Authors:** Qian Qin, Jiayue Huang, Liaojuan Huang, Shenglin Liang, Pingying Zhang, Yuxueyun Li, Ling Tao, Qiaoying Wei

**Affiliations:** 1 The People's Hospital of Guangxi Zhuang Autonomous Region and Guangxi Academy of Medical Sciences, Nanning, China

**Keywords:** smoking cessation, smoking cessation clinic, real-world study, prospective observational study, quit attempts

## Abstract

**INTRODUCTION:**

Smoking cessation clinics are an important component of tobacco control strategies. The study aimed to evaluate the effectiveness of a hospital-based smoking cessation clinic and to examine factors associated with successful smoking cessation in routine clinical practice.

**METHODS:**

A prospective cohort study was conducted at a Smoking Cessation Clinic in China. Smokers who attended the clinic between January 2021 and June 2025 were consecutively enrolled and followed up at 1 month, 3 months, and 6 months. The primary outcome was self-reported continuous abstinence at 6 months. Logistic regression was used to identify factors associated with successful quitting.

**RESULTS:**

A total of 141 smokers were included in the study. The point prevalence abstinence rate was 33.82% at 1 month and 30.28% at 6 months. The rate of quit attempts increased from 73.53% at 1 month to 84.40% at 6 months. The multivariable analysis showed older age was positively associated with successful cessation (adjusted odds ratio, AOR=1.09; 95% CI: 1.01–1.18, p=0.02). Receiving smoking cessation support from parents (AOR=4.06; 95% CI: 1.22–13.52, p=0.02) and other individuals (AOR=6.84; 95% CI: 1.26–37.01, p=0.03) was associated with higher odds of quitting. Compared to smokers who had used smoking cessation drugs, those who had not used medications had lower odds of successful cessation (AOR=0.21; 95% CI: 0.06–0.74, p=0.02), while poorer self-rated health status was also linked to higher odds of abstinence (AOR=4.45; 95% CI: 1.22–16.19, p=0.02).

**CONCLUSIONS:**

In a real-world Chinese cessation clinic, approximately one-third of smokers achieved six-month abstinence. Smoking cessation clinics may help support quit attempts and sustained abstinence among smokers seeking cessation services. Enhancing social support, improving access to pharmacotherapy, and strengthening risk communication may further improve cessation outcomes.

## INTRODUCTION

Tobacco use constitutes a leading cause of preventable death and disease worldwide, representing one of the most critical public health threats^1^. Chinese men consume around 40% of the world’s cigarettes, causing a substantial and growing burden of tobacco-attributed death and disease^2^. Tobacco use is estimated to account for more than 1 million annual deaths in China, and the epidemic continues to increase in men^3^. Tobacco use has substantial health and economic burdens on China; the total economic cost of tobacco use increased significantly from 1.40 trillion RMB in 2014 to 2.43 trillion RMB in 2020, representing an average of 2.29% of Gross Domestic Product annually^4^. In addition, tobacco use imposes a heavy burden of disease and economic costs on society, families, and individuals. Empirical studies suggest that smoking increases the risk of catastrophic health expenditure for households and the likelihood of falling into relative poverty^5,6^.

Lack of willpower and intention, tobacco dependence, influence of surrounding smokers or smoking environments, stress from work or life, etc. were considered as the adverse factors leading to failure in quitting smoking^7^. Smoking cessation is difficult, and many people who smoke experience cessation fatigue as a result of multiple failed attempts^8^. Given these challenges, smoking cessation clinics have been widely recognized by numerous countries and regions as a critical channel to support individuals in quitting tobacco use. Singapore’s inpatient smoking cessation program reported a quit rate of 27.6% at six months^9^. Similarly, in Germany, the continuous self-reported 6-month abstinence rate among therapy-uptake smokers in outpatient settings was 26.3%^10^, while in Thailand, although attendance at smoking cessation clinics has increased, only 20–38% of participants successfully quit smoking^11^. A retrospective study in South Korea reported a higher cessation success rate of 39.6% following a 12-week clinic-based program^12^, whereas a cohort study from Washington University School of Medicine found substantially lower tobacco cessation rates among dual users of cigarettes and e-cigarettes, with only 12.5% abstinent at 6 months and 16.8% at 12 months^13^. Singapore’s inpatient smoking cessation program had a quit rate of 27.6%^14^. In China, similar variability has been observed. A study conducted at a cancer hospital in Hunan Province reported a six-month cessation success rate of 31.7%, and Chen et al.^15^ further demonstrated that smokers with moderate to severe nicotine dependence (Fagerström test for nicotine dependence, FTND score ≥4) exhibited a higher willingness to quit than those with mild dependence (FTND ≤3) in a smoking cessation clinic in Ningbo^16^.

Against this backdrop, robust real-world evidence on the effectiveness of smoking cessation clinics remains limited, particularly in low- and middle-income settings and in regions of China with a high smoking prevalence and relatively constrained tobacco control resources. Most existing studies are short-term, retrospective, or conducted under highly controlled trial conditions, which may overestimate cessation success and fail to capture the complexity of routine clinical practice, including heterogeneous patient profiles, varying adherence, and real-world follow-up challenges^12,13,16^.

Therefore, this study aimed to evaluate the real-world effectiveness of a hospital-based smoking cessation clinic using a prospective cohort design from January 2021 to June 2025, focusing on 6-month smoking cessation outcomes and quit attempts among clinic attendees.

## METHODS

### Study design and participants

A prospective observational study was conducted at the Smoking Cessation Clinic of The People’s Hospital of Guangxi Zhuang Autonomous Region, the largest tertiary care center in Guangxi, China. Participants were consecutively enrolled from smokers who consulted the clinic and agreed to undergo cessation interventions with follow-up, between January 2021 and June 2025. The study was approved by the Ethics Committee of The People’s Hospital of Guangxi Zhuang Autonomous Region (Approval No: LW [2026] 006).

In the study, the analysis focused on smokers who completed the 6-month follow-up. Therefore, smoking cessation outcomes were assessed among participants with complete follow-up data. Participants who did not complete the 6-month follow-up were not included in the final analysis. This approach allowed us to examine smoking cessation patterns and correlates among smokers with complete follow-up information. Inclusion criteria were: 1) having smoked at least 100 cigarettes in their lifetime and smoking at least one cigarette daily in the 30 days preceding the survey; 2) voluntary participation with provision of written informed consent; and 3) age ≥15 years. Exclusion criteria were: 1) low compliance, demonstrated by impatience during the survey or failure to complete all questionnaire items; 2) inability to communicate normally; or 3) refusal to participate in subsequent follow-up assessments.

### Data collection

*Survey materials*

Baseline surveys and follow-up management were conducted using standardized forms designed by the Chinese Center for Disease Control and Prevention (China CDC): the smoking cessation clinic registration form, the month 1 follow-up form, the month 3 follow-up form, and the month 6 follow-up form.

*Baseline surveys*

Upon visiting the cessation clinic, the physician collected basic information and guided participants in completing the registration form. This form covered demographic characteristics (age, gender, education level), smoking profile (average daily cigarette consumption, years of smoking, nicotine dependence level, etc.), cessation-related characteristics (social support for quitting, use of cessation medications, etc.), and health status. Nicotine dependence was assessed using the Fagerström test for nicotine dependence (FTND)^17^. The total score ranges from 0 to 10, with higher scores indicating greater severity of dependence. Scores are categorized as: low (0–3), moderate (4–6), or severe (7–10) dependence. Smoking exposure was assessed using the Smoking Index (SI), calculated as the product of the average number of cigarettes smoked per day and the number of years of smoking. Higher SI values indicate greater cumulative smoking exposure. The SI was categorized as: low (0–199), moderate (200–400), or severe (>400).

*Follow-up surveys*

Trained nurses followed up participants who completed the baseline survey and entered the cessation program via telephone. Follow-ups were conducted in accordance with the respective follow-up forms at 1 month, 3 months and 6 months. Participants were considered lost to follow-up if they could not be contacted after five or more call attempts at different times. Those who were contacted but refused to participate were recorded as refusals. Quit attempt was defined as abstinence for ≥24 hours.

### Measures

*Successful smoking cessation*

Smoking status during follow-up was based on self-report. Successful smoking cessation was defined as reporting no smoking of any tobacco products, including conventional cigarettes and e-cigarettes, from the initial clinic visit until the follow-up assessment. In the surveys, participants were asked: ‘Have you smoked any cigarettes, even one or two puffs, since your first clinic visit?’. For this study, the primary outcome variable was successful smoking cessation at the follow-up at 6 months, coded as 1 (‘yes’) or 0 (‘no’). Using the month 6 time point to determine cessation success is a method employed in multiple prior studies^18-20^. The point-prevalence abstinence rate was calculated as: (Number of successful quitters at the time point/Total number of participants who completed that follow-up) × 100%. It is worth noting that participants lost to follow-up were conservatively classified as smokers in a sensitivity analysis.

*Smoking cessation attempts*

Participants were asked during follow-up: ‘During the follow-up period, have you tried to quit smoking (achieving abstinence for ≥24 hours)?’. A ‘yes’ response was defined as having made a quit attempt. The point prevalence rate of quit attempts was calculated as: (Number reporting a quit attempt at the time point/Total number of participants completing that follow-up) × 100%, again excluding those lost to follow-up or who refused.

### Covariates

Sociodemographic covariates included age (≤ 30, 31–40, 41–50, 51–60, and ≥61 years), gender (male, female), education level (primary school or lower, junior high school, high school/secondary specialized school, junior college, Bachelor’s degree or higher), and family monthly income (<3000, 3000–4999, 5000–6999, 7000–8999, ≥9000 RMB, or not sure).

Cessation support and health behavior covariates included sources of smoking cessation support (from parents, children, or other individuals; each as yes, no), use of smoking cessation drugs (yes, no), and self-perceived health status (good, poor).

Smoking characteristics and exposure covariates included nicotine dependence assessed by the FTND score (categorized as low, moderate, or severe), cumulative smoking exposure measured by the Smoking Index (categorized as low, moderate, or severe), and the exhaled carbon monoxide level at baseline (categorized as low <10 ppm or high ≥10 ppm).

### Statistical analysis

Descriptive statistics were used to summarize the characteristics of the study population. Categorical variables are presented as frequencies (n) and percentages (%). Multivariable logistic regression was performed to examine factors associated with successful smoking cessation at 6 months, using successful cessation as the dependent variable. All covariates described above were included in the model as potential confounding variables. The independent variables included age, gender, education level, family monthly income, sources of smoking cessation support (from parents, children, or other individuals), use of smoking cessation drugs, self-perceived health status, nicotine dependence assessed by the FTND score, cumulative smoking exposure measured by the Smoking Index, and baseline exhaled carbon monoxide level. The results are presented as adjusted odds ratios (AORs) and 95% confidence intervals (CIs). Data cleaning and analysis were performed using STATA software (version 18.0). A p<0.05 was considered statistically significant.

## RESULTS

### Demographic characteristics

A total of 141 smokers were enrolled in the study, including 132 males (93.62%). The mean age of smokers was 42.56 years. Smoking cessation support was most commonly provided by parents (56.74%, n=80), followed by children (39.72%, n=56) and other individuals (12.06%, n=17). Among the participants, 51.77% of smokers (n=53) reported having used smoking cessation medications during their quit attempt. In addition, 74.47% of participants (n=105) rated their self-perceived health status as good. With respect to nicotine dependence, as measured by the FTND, 35.46% of smokers (n=50) were categorized as having low nicotine dependence, while 41.13% (n=58) fell into the moderate dependence category. Regarding cumulative smoking exposure, assessed using the Smoking Index (SI), 21.28% of smokers (n=30) were classified as having low smoking exposure, whereas 43.97% (n=58) were categorized as having moderate exposure.

### Smoking cessation characteristics

Regarding smoking cessation behaviors during the follow-up period, the number of smokers who reported smoking between baseline and follow-up gradually declined over time (Table 2). Similarly, the number of individuals who reported no smoking during this period also decreased. Most smokers made at least one quit attempt during follow-up. The number of individuals reporting a quit attempt decreased from 100 at follow-up at 1 month to 92 at 6 months. However, some smokers who initially achieved abstinence for ≥24 hours subsequently resumed smoking.

**Table 1:** Baseline demographic and smoking-related characteristics of study participants, Guangxi, China, 2021–2025 (N=141)

*Characteristics*	*n (%)*
**Age** (years)	
≤30	20 (14.18)
31–40	55 (39.01)
41–50	32 (22.7)
51–60	20 (14.18)
≥61	14 (9.93)
**Gender**	
Male	132 (93.62)
Female	9 (6.38)
**Smoking cessation support from parents**	
No	80 (56.74)
Yes	61 (43.26)
**Smoking cessation support from children**	
No	85 (60.28)
Yes	56 (39.72)
**Smoking cessation support from other individuals**	
No	124 (87.94)
Yes	17 (12.06)
**Smoking cessation medications use**	
No	68 (48.23)
Yes	73 (51.77)
**Self-perceived health status**	
Good	105 (74.47)
Poor	36 (25.53)
**Exhaled carbon monoxide**	
Low	134 (95.04)
High	7 (4.96)
**Family monthly income (RMB)**	
<3000	14 (9.93)
3000–4999	17 (12.06)
5000–6999	23 (16.31)
7000–8999	27 (19.15)
≥9000	50 (35.46)
Not sure	10 (7.09)
**FTND level**	
Low	50 (35.46)
Moderate	58 (41.13)
Severe	33 (23.4)
**Smoking index**	
Low	30 (21.28)
Moderate	62 (43.97)
Severe	49 (34.75)
**Education level**	
Primary school and lower	8 (5.67)
Junior high school	27 (19.15)
High school/secondary specialized school	30 (21.28)
Junior college	23 (16.31)
Bachelor’s degree or higher	53 (37.59)

RMB: 1000 Chinese Renminbi about US$150

**Table 2:** Smoking cessation characteristics at follow-up at 1 month, 3 months, and 6 months, among smokers enrolled in the cessation clinic, Guangxi, China, 2021–2025 (N=141)

*Variables*	*1 month*	*3 months*	*6 months*
Smoked between baseline and follow-up	90	84	76
No smoking between baseline and follow-up	46	35	33
Attempted to quit smoking during follow-up (abstinence ≥24 h)	100	95	92
Smoked daily during quit attempt (if not abstinent)	22	26	30
6-month follow-up completers	136	119	109
Lost to follow-up	5	17	10
Total	141	136	109

**Figure 1:**
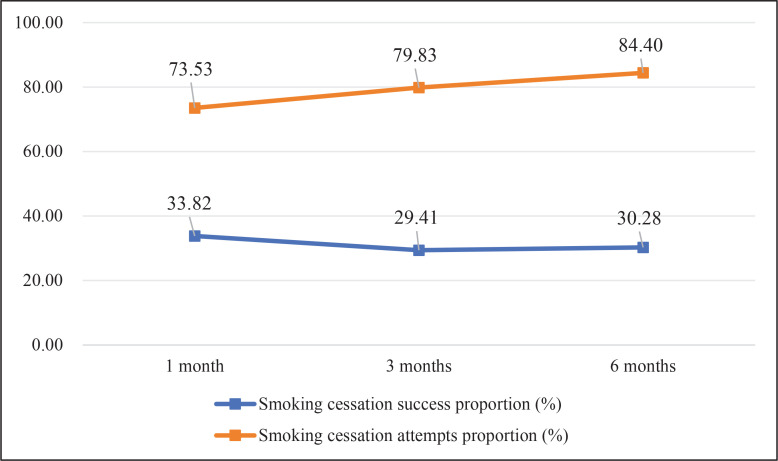
Point prevalence abstinence rates and quit attempt rates at follow-up at 1 month, 3 months, and 6 months, among smokers enrolled in the cessation clinic, Guangxi, China, 2021–2025 (N=141, 136, 109, respectively)

Figure 1 presents the rates of successful smoking cessation and quit attempts at different time points. The cessation rate (defined as no smoking between baseline and follow-up) decreased from 33.82% at 1 month to 30.28% at 6 months. The quit attempt rate increased from 73.53% at 1 month to 84.40% at 6 months.

**Figure 2:**
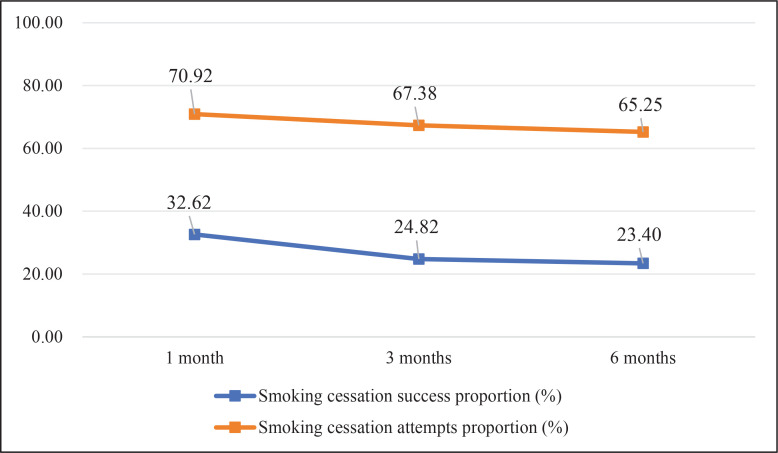
Point prevalence abstinence rates and quit attempt rates at follow-up at 1 month, 3 months, and 6 months, among smokers enrolled in the cessation clinic, Guangxi, China, 2021–2025 (N=141)

In addition to the completer-only analysis, we conducted a sensitivity analysis assuming that all participants lost to follow-up at 6 months were considered as smokers. As shown in Figure 2, the 6-month abstinence rate decreased from 32.62% to 23.40%. The overall pattern of associations remained similar to the primary analysis. The trend in quit attempts showed slight differences, with the rate decreasing from 70.92% at 1 month to 65.25% at 6 months.

### Factors associated with successful smoking cessation

Only participants who completed the 6-month follow-up were included in the multivariable logistic regression analysis. Participants who did not complete the 6-month follow-up were excluded from the analysis.

Table 3 displays the results of the multivariable logistic regression analysis for successful smoking cessation. Age was positively associated with successful cessation (AOR=1.09; 95% CI: 1.01–1.18, p=0.02). Smoking cessation support from parents (AOR=4.06; 95% CI: 1.22–13.52, p=0.02) and from other individuals (AOR=6.84; 95% CI: 1.26–37.01, p=0.03) was associated with significantly higher odds of successful quitting. Compared to smokers who had used smoking cessation drugs, those who had not used medications had lower odds of successful cessation (AOR=0.21; 95% CI: 0.06–0.74, p=0.02). Smokers with poorer self-rated health had higher odds of successful cessation (AOR=4.45; 95% CI: 1.22–16.19; p=0.02).

**Table 3:** Multivariable logistic regression analysis of factors associated with successful smoking cessation at follow-up at 6 months among smokers attending a smoking cessation clinic, Guangxi, China, 2021–2025 (N=109)

*Variables*	*AOR*	*SE*	*z*	*p*	*95% CI*	
					*Lower*	*Upper*
**Age** (years)	1.09	0.04	2.29	0.02	1.01	1.18
**Gender** (ref. male)						
Female	0.88	0.96	-0.12	0.91	0.10	7.48
**Smoking cessation support from parents** (ref. no)						
Yes	4.06	2.49	2.28	0.02	1.22	13.52
**Smoking cessation support from children** (ref. no)						
Yes	0.54	0.34	-0.98	0.33	0.15	1.87
**Smoking cessation support from other individuals** (ref. no)						
Yes	6.84	5.89	2.23	0.03	1.26	37.01
**Smoking cessation medications use** (ref. yes)						
No	0.21	0.14	-2.43	0.02	0.06	0.74
**Self-perceived health status** (ref. good)						
Poor	4.45	2.93	2.26	0.02	1.22	16.19
**Exhaled carbon monoxide** (ref. low)						
High	2.97	3.89	0.83	0.41	0.23	38.78
**Family monthly income** (ref. <3000)						
3000–4999	0.73	0.81	-0.29	0.77	0.08	6.40
5000–6999	2.50	2.65	0.87	0.39	0.31	19.97
7000–8999	0.90	0.89	-0.11	0.92	0.13	6.31
≥9000	1.50	1.45	0.42	0.68	0.23	9.99
Not sure	2.58	3.41	0.71	0.48	0.19	34.54
**FTND level** (ref. low)						
Moderate	1.48	0.90	0.65	0.52	0.45	4.87
Severe	0.91	0.64	-0.14	0.89	0.23	3.63
**Smoking Index** (ref. low)						
Moderate	1.67	1.24	0.69	0.49	0.39	7.19
High	0.68	0.69	-0.38	0.70	0.09	4.96
Constant	0.04	0.08	-1.66	0.10	0.01	1.80

AOR: adjusted odds ratio. The adjusted odds ratios were estimated using a multivariable logistic regression model controlling for potential confounding variables. SE: standard error. FTND: Fagerström test for nicotine dependence.

## DISCUSSION

This prospective cohort study with six-month follow-up, was conducted in a real-world smoking cessation clinic in Southwest China. The results showed that approximately one-third of smokers achieved self-reported continuous abstinence at six months.

The 6-month cessation rate observed in our study is notably higher than that reported among dual users of cigarettes and e-cigarettes in a US cohort. It may be associated with the difference in outcome definitions. In previous studies, participants who are lost to follow-up are conservatively classified as smokers when calculating abstinence outcomes. In contrast, the present study focused on participants who completed the 6-month follow-up, and cessation outcomes were assessed among those with complete follow-up data. The outcome definitions should be considered when comparing our findings with those of other studies. Moreover, dual users often exhibit higher nicotine dependence and face greater challenges in achieving abstinence compared to exclusive cigarette smokers seeking treatment in a dedicated clinic^21,22^. Within the Chinese context, the findings are consistent with prior evidence; the observed quit rate aligns with previous reports from hospital-based cessation programs in Hunan Province, suggesting a probably similar magnitude of effect for dedicated cessation clinics in Chinese hospitals. Importantly, the gradual decline in cessation rates from 1 month to 6 months reflects the chronic, relapsing nature of nicotine dependence rather than failure of the intervention per se^23^. Many participants achieved short-term abstinence but subsequently relapsed, a pattern widely documented in cessation research^24,25^. The sensitivity analysis using the conservative assumption that participants lost to follow-up were smokers yielded slightly lower abstinence rates but did not materially change the observed associations.

Beyond sustained abstinence, this study revealed a high prevalence of quit attempts throughout the follow-up period, with most participants reporting at least one attempt at each time point. Quit attempts are widely regarded as a critical intermediate outcome in smoking cessation, as repeated attempts substantially increase the probability of eventual success^26^. The high rate of attempts observed here suggests that clinic-based interventions may be effective in initiating quitting behavior and maintaining engagement with cessation efforts.

The gradual decline in quit attempts over time likely reflects diminishing motivation, withdrawal fatigue, or negative affect etc.^27,28^. Nonetheless, this pattern is consistent with existing literature and reinforces the need to conceptualize smoking cessation as a dynamic, iterative process rather than a single binary outcome^29^. From this perspective, cessation clinics play an important role not only in producing immediate abstinence but also in sustaining smokers’ willingness to reattempt quitting after relapse.

Several factors were independently associated with successful cessation at six months. Older age was positively associated with quitting, a finding consistent with prior studies suggesting that older smokers may perceive greater health risks or experience stronger external pressures to quit, due to accumulating comorbidities or family responsibilities^30,31^. Support from parents and other individuals was associated with markedly higher odds of quitting. In the Chinese sociocultural context, family influence and interpersonal obligations may amplify the impact of social support on health-related behaviors^7^. Use of smoking cessation medications was also strongly associated with successful abstinence. Participants who did not use pharmacotherapy had substantially lower odds of quitting, reinforcing existing evidence that medication-assisted cessation is a key component of effective treatment^32^. Despite strong guideline recommendations^33^, pharmacotherapy remains underutilized in many real-world settings in China, often due to limited access, the self-payment for smoking cessation medications, or insufficient awareness^34^. Interestingly, poorer self-rated health status was associated with higher odds of successful cessation. This finding likely reflects heightened risk perception or symptom-driven motivation among smokers experiencing health deterioration^35^, consistent with health-related behavior theories emphasizing perceived vulnerability as a trigger for behavior change^36^.

### Recommendations

Based on the findings of this study, the following recommendations are proposed. First, enhancing social and family support may help smokers maintain motivation during the quitting process. Second, improving access to evidence-based pharmacological treatments may support smokers in managing nicotine dependence and withdrawal symptoms. Finally, strengthening risk communication about the health consequences of smoking may further encourage quit attempts and sustained abstinence. However, given the observational nature of this study without a control group, these implications should be interpreted with caution and warrant confirmation in future controlled studies.

### Strengths and limitations

This study has several strengths, including its prospective design, standardized data collection using nationally endorsed instruments, and an extended observation period within a real-world clinical setting.

However, several limitations should be acknowledged. Smoking status was self-reported and not biochemically verified, which may have led to overestimation of cessation rates. Moreover, although we adjusted for several sociodemographic and smoking-related factors in the multivariable analysis, residual confounding from unmeasured or inadequately measured variables cannot be entirely ruled out. Loss to follow-up may have introduced selection bias, and the single-center design with a relatively small sample size may limit generalizability.

## CONCLUSIONS

This prospective real-world study demonstrates that clinic-based smoking cessation services in China may promote high levels of quit attempts and achieve sustained abstinence in a meaningful proportion of smokers. Social support, pharmacotherapy use, age, and perceived health status are important correlates of cessation success. Future studies with controlled designs and objective outcome measures are needed to confirm these observations and to provide sufficient evidence for policy development.

## Data Availability

The data supporting this research are available from the authors on reasonable request.
